# Hydroxycinnamic Acids and Their Derivatives: Cosmeceutical Significance, Challenges and Future Perspectives, a Review

**DOI:** 10.3390/molecules22020281

**Published:** 2017-02-13

**Authors:** Oludemi Taofiq, Ana M. González-Paramás, Maria Filomena Barreiro, Isabel C. F. R. Ferreira

**Affiliations:** 1 Mountain Research Centre (CIMO), ESA, Polytechnic Institute of Bragança, Campus de Santa Apolónia, 1172, 5300-253 Bragança, Portugal; taofiq.oludemi@ipb.pt; 2 Grupo de Investigación en Polifenoles (GIP), Unidad de Nutrición y Bromatología, Faculty of Pharmacy, University of Salamanca, Campus Miguel de Unamuno, 37007 Salamanca, Spain; paramas@usal.es; 3 Laboratory of Separation and Reaction Engineering (LSRE), Associate Laboratory LSRE/LCM, Polytechnic Institute of Bragança, Campus de Santa Apolónia, 1134, 5301-857 Bragança, Portugal; barreiro@ipb.pt

**Keywords:** cosmetics, hydroxycinnamic acids, skin care, microencapsulation

## Abstract

Bioactive compounds from natural sources, due to their widely-recognized benefits, have been exploited as cosmeceutical ingredients. Among them, phenolic acids emerge with a very interesting potential. In this context, this review analyzes hydroxycinnamic acids and their derivatives as multifunctional ingredients for topical application, as well as the limitations associated with their use in cosmetic formulations. Hydroxycinnamic acids and their derivatives display antioxidant, anti-collagenase, anti-inflammatory, antimicrobial and anti-tyrosinase activities, as well as ultraviolet (UV) protective effects, suggesting that they can be exploited as anti-aging and anti-inflammatory agents, preservatives and hyperpigmentation-correcting ingredients. Due to their poor stability, easy degradation and oxidation, microencapsulation techniques have been employed for topical application, preventing them from degradation and enabling a sustained release. Based on the above findings, hydroxycinnamic acids present high cosmetic potential, but studies addressing the validation of their benefits in cosmetic formulations are still scarce. Furthermore, studies dealing with skin permeation are scarcely available and need to be conducted in order to predict the topical bioavailability of these compounds after application.

## 1. Introduction

According to the European Union (EU) Cosmetics Regulation [1], a “cosmetic product” means any substance or mixture intended to be placed in contact with the external parts of the human body (epidermis, hair system, nails, lips and external genital organs) or with the teeth and the mucous membranes of the oral cavity, exclusively, or mainly, cleaning, perfuming, changing appearance, protecting, maintaining good condition or correcting body odors.

The cosmetic industry is a growing industrial sector where, according to financial statistics, the European cosmetic industry is worth 70 billion euros [2]. Its global market is divided into several categories, such as skin, hair, color, fragrance and odor care [3]. Due to consumer’s demands and environmental limitations, this industry is in continuous search for new ingredients, namely the ones obtained from natural sources with environmentally-friendly connotations and less toxic properties [4]. These options offer the advantage to replace synthetic counterparts, sometimes facing restrictions according to the applied regulations to assess the risk to human health and the environment [2]. Natural ingredient, such as phytonutrients, microbial metabolites, dairy products, minerals and animal protein components, are becoming increasingly applied in cosmeceutical formulations and taken orally to produce a positive appearance benefit [5].

Plant-derived compounds have been reported for cosmeceutical applications. Furthermore, attention has been directed to the waste and residue produced during their processing, resulting in some of their derived bio-products, which may have the potential to be applied as cosmetic ingredients [6]. Bio-products of plant species, such as citrus, tomato and olives, contain compounds like limonene, lycopene, unsaturated fatty acids and phenolic compounds that have shown biological properties, such as antioxidant, anti-inflammatory and anti-tyrosinase activity. The authors suggest that further studies need to be conducted with other food by-products to attribute them a place in the cosmetic sector. Rodrigues et al. [7] reported that different bio-products of the olive oil processing industry (leaves, stones, waste water and pomace) displayed high antioxidant activity and presented bioactive compounds, such as minerals and fatty acids, with functional roles compatible with cosmetic ingredients. Hydroxy acids, such as glycolic, hydroxybutanoic, malic, citric, salicylic and lactic acids, isolated from several natural sources have been highlighted in the skin care sector for anti-wrinkling benefits, radical-scavenging effects and treatment of several hyperpigmentation disorders. Their safety is constantly evaluated, and among them, glycolic and salicylic acids are becoming very popular in skin care products [8].

In a recent work, Taofiq et al. [5] reviewed some relevant studies carried out with mushrooms and their metabolites as important cosmetic ingredients. The authors reported that the extracts, as well as their bioactive metabolites showed promising anti-tyrosinase, anti-hyaluronidase, anti-collagenase and anti-elastase activity, besides the well-documented anti-oxidant, antimicrobial and anti-inflammatory properties. They also identified the need to conduct more studies in dermal and epidermal cells in order to determine the mechanism of action of some of these mushroom metabolites. Moreover, mushroom bioactive ingredients should be incorporated in cosmetic matrices and tested to estimate their effectiveness to cross the skin barrier. Following these trends, the studies of Taofiq et al. [9] examined the incorporation of mushroom ingredients into a base cosmetic cream. The prepared cosmeceutical formulation was found to retain considerable amounts of phenolic acids and ergosterol, displaying antioxidant, anti-inflammatory, anti-tyrosinase and antibacterial activity.

Also in the context of cosmetic ingredients, Wang et al. [10] reviewed the bioactive properties of marine algae. The authors highlighted the presence of alginate and mycosporine-like amino acids (MMAs) imparting anti-oxidative properties and the ability to act as reactive oxygen species (ROS) scavengers. Some algae extracts were also reported to inhibit activator protein (AP-1) and nuclear factor kappa β (NF-κβ), both transcription factors associated with the expression of enzymes, like matrix metalloproteinase and inducible nitric oxide synthase responsible for collagen degradation, and to increase the release of inflammatory mediators causing skin wrinkling and inflammation, respectively. The authors also mentioned the potential of bioactive compounds, such as fucoxanthin (carotenoid), phlorotannins and phloroglucinol (phenol derivative), to inhibit the tyrosinase enzyme and prevent melanin biosynthesis. These multiple properties displayed by marine algae and their bioactive components have demonstrated that they can be exploited as new and powerful cosmeceutical ingredients.

Animal processing for human consumption also generates a handful of residues and raw materials, such as collagen, elastin and keratin, ingredients that are slowly finding their way into the cosmetic industry. Collagen is often recovered from the hides, skin and bones of cows and pigs, while elastin has been recovered from the ligaments of cow, pork, fish and chicken. They are both important components of the extracellular matrix, being responsible for both skin elasticity and flexibility maintenance. Keratin, on the other hand, can be recovered from poultry feathers and porcine hairs. In combination, elastin and collagen offer the mechanical resistance necessary for an intact skin [11]. Marine animals, such as starfish, sponges and jellyfish, are other raw material sources with growing importance in the cosmetic industry due to their richness in bioactive compounds, such as phenolics and carotenoids [12]. The mentioned authors conducted comprehensive studies to evaluate the antioxidant, anti-collagenase, anti-elastase and anti-hyaluronidase activity of stalked sea squirt (*Styela clava* Herdman) extract. The results revealed that the obtained extract inhibited collagenase, elastase and hyaluronidase enzymes, highlighting its potential to maintain the flexibility and elasticity of the skin, as well as moisture content and tension. The compounds responsible for the shown activity were not identified or isolated. Earthworm (*Lumbricus terrestris* Linnaeus) has also been reported to show cosmeceutical significance even though very few studies have been reported so far [13]. These authors described elastase, tyrosinase and collagenase inhibitory activities, suggesting that the extract contains useful anti-wrinkling and depigmenting ingredients.

All of these interesting reports have shown that extracts and compounds obtained from diverse natural sources have high potential to be used as cosmetic ingredients and thus should be investigated for this purpose. In particular, very few studies have been carried out to determine the mechanism of action of the relevant extracts, as well as to identify the individual bioactive metabolites responsible for the ascribed bioactivities.

Phenolics can be divided into several groups, such as phenolic acids, flavonoids, tannins, stilbenes and lignans, among others [14]. All of these compounds have been extracted from various natural matrices with the help of organic solvents and conventional procedures (maceration, Soxhlet extraction), ultrasound and microwave-assisted extractions. These procedures can be rather ineffective in extracting most of the phenolic compounds, driving an increased attention to develop/apply better and more efficient extraction procedures [15]. Among phenolics, phenolic acids are the most common and ubiquitous bioactive compounds found in plants, fungi, marine algae and other natural sources. There are two major groups of phenolic acids, the hydroxycinnamic acids and the hydroxybenzoic acid groups [16].

According to Dr. Albert Kligman, who is often regarded as the father of cosmeceuticals, a cosmeceutical ingredient is evaluated if three inquiries are answered. Firstly, can the ingredient cross the stratum corneum (SC) of the skin and be available at its desired concentration? Secondly, does the ingredient have a specific mechanism of action in dermal or epidermal cells or tissues? Lastly, are there published or peer reviewed clinical trials to support that the ingredient works as claimed [17]. In the present review, the cosmeceutical significance of hydroxycinnamic acids and their derivatives will be evaluated seeking for answers to the above questions in the literature.

## 2. Hydroxycinnamic Acids

**Figure 1:**
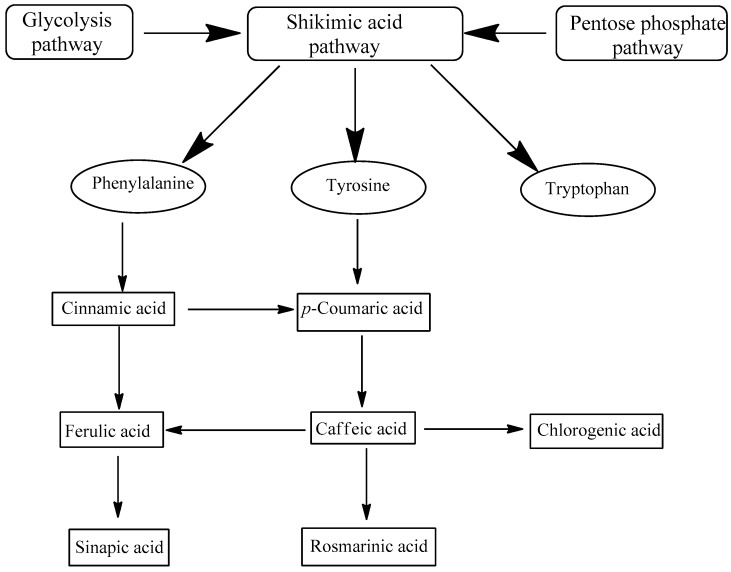
Schematic representation of hydroxycinnamic acid synthesis.

**Figure 2:**
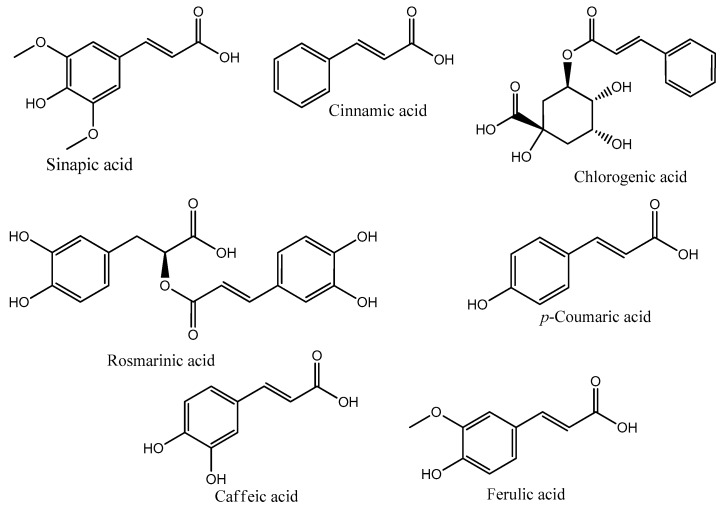
Hydroxycinnamic acids and their derivatives.

Phenolic compounds are a group of secondary metabolites from fungi and plants, not needed for growth and reproduction. They are secreted for protection against UV, competitive warfare against other plants, insects, viruses and bacteria, being also responsible for plant smell, color and flavor [18]. Phenolic acids are derived from the shikimate pathway involving several enzymatic steps responsible for converting intermediates of the pentose phosphate pathway and glycolysis to form three crucial aromatic amino acids (phenylalanine, tyrosine and tryptophan) [16,18] (Figure 1). Phenylalanine and tyrosine give rise to two major groups of phenolic acids: (i) the hydroxycinnamic acids group (Figure 2), the largest class, comprises a three-carbon side chain (C6–C3) structure; examples include caffeic, ferulic, *p*-coumaric and sinapic acids; (ii) the hydroxybenzoic acids group comprises a C6–C1 structure; it includes gallic, *p*-hydroxybenzoic, protocatechuic, vanillic and syringic acids [19].

Hydroxycinnamic acids can be also found in derivative forms, such as amides (combination with amino acids or peptides) and esters (combination with hydroxyl acids or glycosides). These forms have also been reported for their biomedical and industrial potential, but very few data are available to support their application as cosmeceutical ingredients [20]. They exhibit multiple physiological functions, such as antioxidant, anti-inflammatory, anti-microbial, anti-collagenase and anti-melanogenic activity; properties that are the basis of the driving force behind a gradual increased use of hydroxycinnamic acids themselves and their derivatives in skin care cosmetic formulations. The pharmacological potential displayed by these phenolic acids and derivatives has been largely attributed to the presence of multiple hydroxyl groups in their chemical structure, making them suitable free radical scavengers [21].

*p*-Coumaric acid is a phenolic acid synthesized mainly from tyrosine and phenylalanine. It is a major precursor in the synthesis of other phenolic acids, such as caffeic, chlorogenic, rosmarinic and ferulic acids. It is widely distributed in fruits, vegetables, cereals and mushrooms [22,23]. Studies on *p*-coumaric acid and its conjugated forms revealed properties, such as antioxidant, antimicrobial, antitumor, anti-inflammatory, antiplatelet aggregation, as well as other interesting health benefits [24]. Among the above-mentioned properties, their depigmenting potential, antioxidant, anti-collagenase, antimicrobial and anti-inflammatory activities seem to be the most important for cosmeceutical use [25].

Caffeic acid is one of the most common phenolic acids found in fruits, vegetables, mushrooms and herbs. It is biosynthesized by hydroxylation of *p*-coumaric acid and has medicinal properties, such as antioxidant, antitumor, anti-inflammatory, antimicrobial and antidiabetic activity [18,26]. Ferulic acid, 4-hydroxy-3-methoxycinnamic acid, is widely distributed in beverages (coffee, beer), fruits (cabbage, potatoes, carrots), vegetables (broccoli, spinach, tomato), cereals (wheat, corn, maize), flowers and nuts [15,27]. It is a caffeic acid derivative formed by the action of the enzyme caffeate *O*-methyltransferase. It has well-documented antioxidant, antitumor, UV-absorbing and anti-inflammatory activities, and great attention is now directed toward its incorporation into cosmetic emulsions for topical application [28].

Rosmarinic acid is found in *Rosmarinus officinalis* L., *Salvia officinalis* L., *Mentha piperita* L. and *Thymus vulgaris* L.; it results from both caffeic acid and 3,4-dihydroxyphenyllactic acid esterification [29]. It has interesting antioxidant, antitumor, anti-inflammatory and antimicrobial properties [29,30]. Because of its high radical-scavenging activity and other reported medicinal benefits, it is attracting interest from the pharmaceutical and cosmetic sectors [31]. Chlorogenic acid, obtained from the esterification of caffeic acid and l-quinic acid (3-caffeoylquinic acid), is widely distributed in coffee, apples and pears and is one of the most important hydroxycinnamic acid derivatives in plants. There are numerous publications that support the potential of this compound as an anti-inflammatory, antidiabetic, antiviral, antioxidant and anti-tyrosinase agent [32,33]. Sinapic acid, with reported antioxidant and anti-inflammatory properties, is also present in fruits and vegetables. It is formed from methoxyl and hydroxyl substitution of caffeic acid, to form intermediately ferulic acid, which is further methylated to form sinapic acid. Due to the presence of methoxyl and hydroxyl groups in its structure, sinapic acid has high radical-scavenging activity [34].

### 2.1. Anti-Aging and Depigmentation Properties

Oxidative stress, due to ROS generation, plays a major role in aging and other related diseases associated with damages to biomolecules like DNA, lipids and proteins [35]. The intrinsic pool of enzymatic antioxidants, such as superoxide dismutase (SOD), catalase and glutathione peroxidase (GP), which neutralize free radicals and repair oxidative damage, is highest in human skin [36], but is often insufficient. This supports the search for natural antioxidants to serve as an exogenous source to compensate the high level of free radical generation in the body [37].

The skin can be considered as a network of cellular and non-cellular (extra-cellular matrix) components, like collagen, elastin, glycoproteins and hyaluronic acid. It controls cellular processes occurring in the skin and also acts as a well-equipped defense system against skin damages. All of its components play functional roles to maintain the skin in the proper condition. Nevertheless, with age, their levels decrease as a consequence of several biochemical processes occurring in the body resulting in various clinical manifestations associated with aging [38,39]. In this context, the use of extracts and their bioactive metabolites as topical ingredients has proven to be effective as photoprotectors against UV radiation and as immune suppressors of the NF-κβ pathway [40]. In general, antioxidants act as metal chelators, lipid peroxidation inhibitors and ROS scavengers and are incorporated in cosmetic formulations to reduce aging effects. As such, they can be effective as anti-wrinkle and depigmentation ingredients, thus preventing damage from UV radiation, whose effect leads to ROS generation, increasing the expression of matrix metalloproteinase 1 (MMP-1) and tyrosinase, both enzymes responsible for collagen breakdown and hyperpigmentation, respectively [5].

Tyrosinase acts in the rate-limiting step of the biosynthesis of melanin. Several extracts, as well as bioactive compounds have been found to block this pathway and reduce the formation of melanin. The mechanism of action has not been fully understood, but these compounds usually block the microphthalmia-associated transcription factor (MITF) responsible for the expression of tyrosinase enzyme [5]. The EU cosmetic market is a dynamic market that has monitored the use of hyperpigmentation-correcting ingredients and, among them, corticosteroids, hydroquinone, monobenzyl hydroquinone, tretinoin and mercury salts have been banned by the EU Cosmetic Regulation [1]. Furthermore, this industry is constantly searching for alternative ingredients with less side effects, and substances like kojic acid, ascorbic acid, arbutin, lactic acid, salicylic acid and nicotinamide, among others, are now common hyperpigmentation agents allowed in the EU cosmetic market [41,42].

Other studies have been conducted on the anti-collagenase, photoprotective, anti-hyaluronidase and anti-tyrosinase activities of hydroxycinnamic acids and their derivatives, making them very important cosmeceutical ingredients (Table 1).

Among the various classes of antioxidants, phenolic compounds are the most important ones showing effective protection against UV, thus proving their ability to be used in sunscreen formulations [63]. Saija et al. [48] stated that the use of compounds with radical scavenging activity can be very effective to reduce UV-induced skin aging. The authors were among the first ones conducting studies on the potential of commercial ferulic and caffeic acids as protective ingredients against UV-induced skin aging, which included also testing on human skin samples. They evaluated the photoprotective effect of both compounds in UVB-exposed skin cells and conducted skin permeation studies using the Franz diffusion apparatus to estimate the topical availability of both compounds. Both molecules were found to offer photoprotection to UVB-induced skin erythema relative to the control. Even ferulic acid was reported to permeate the skin better than caffeic acid, which was justified by its higher lipophilicity; both compounds were found adequate to be used as topical UV filters in cosmetic formulations to prevent photo-aged skin. Psotova et al. [58] isolated rosmarinic acid from a hydroethanolic extract of *Prunella vulgaris* L. and evaluated its ability to offer a photoprotective effect on human keratinocytes cells exposed to UVA radiation. After incubating the cells with 0.9–18 mg/L rosmarinic acid, UVA-induced changes in human keratinocytes cells were suppressed, suggesting that this compound should be further investigated as a novel skin photoprotective material.

Pluemsamram et al. [47] evaluated the anti-collagenase activity of ferulic (FA) and caffeic acid (CA)-based on their ability to suppress MMP-1 activity in UVA-induced human keratinocyte (HaCaT) cells and also their photoprotective ability by measuring antioxidant defense parameters. FA at (15–30 µM) and CA at (3.75–30 µM) suppressed UVA-induced MMP-1 activity and restored antioxidant parameters, suggesting that both compounds can act as anti-wrinkling and photoprotective ingredients for skin care products. Seok et al. [55] evaluated the potential of *p*-coumaric acid to inhibit MMP-1 activity in human dermal fibroblasts HaCaT cells exposed to UVB radiation. At 30 µg/mL, MMP-1 expression from dermal fibroblasts was inhibited, implying that this compound has the ability to prevent collagen degradation and improve the tensile strength and skin elasticity.

Antioxidant and antimelanogenic activity are very important properties in the design of cosmetic products, and as such, Kwak et al., 2011 [43], evaluated the potential of synthesized caffeic acid conjugated with an amino acid as a cosmetic ingredient by testing its anti-tyrosinase and antioxidant activities. The conjugated compound caffeoyl-amino acidyl-hydroxamic acid was found to display radical scavenging activity and to act as a lipid peroxidation inhibitor. At 100 µM, the compound significantly inhibited tyrosinase activity without a cytotoxic effect. Both results proved the cosmeceutical potential of the compound, which can be further analyzed against other related bioactivities. Kwak et al., 2013 [64], studied caffeic, *p*-coumaric, ferulic and sinapic (SA) acids and their synthesized derivatives, considering antioxidant activity and tyrosinase inhibition, to be used as ROS scavengers and improve hyperpigmentation. The antioxidant results showed that CA, FA and SA derivatives displayed a radical-scavenging activity higher than their parent compounds and trolox, used as the positive control. CA derivatives were also found to be the most interesting inhibitors of tyrosinase in melanocyte cells and, as such, can be vital ingredients in the design of cosmetic formulations against aging and hyperpigmentation.

Georgiev et al. [65] evaluated the anti-tyrosinase, antioxidant and antimicrobial activities of synthesized derivatives of ferulic, caffeic, sinapic and *p*-coumaric acids. All of the tested compounds showed DPPH (2,2-diphenyl-1-picrylhydrazyl) radical scavenging activity, as well as the capacity to inhibit lipid peroxidation. The compounds displayed antibacterial activity against *Staphylococcus aureus* and *Streptococcus pyogenes*. The authors also reported anti-tyrosinase activity for these compounds and went on to suggest that the structural similarity among hydroxycinnamic acids and tyrosine may be linked to the observed activity. The multiple biological properties exhibited by these compounds show that they should be further studied as cosmetic ingredients. The continuous search for more effective hyperpigmentation-correcting alternatives, with less toxic effects and better properties, has led Song et al. [56] to evaluate the antimelanogenic potential of *p*-coumaric acid (PCA) and its methyl ester (MPA) in human epidermal melanocytes based on their ability to inhibit the tyrosinase enzyme and halt melanin biosynthesis. The potential of both compounds to penetrate the skin was also evaluated. The results showed that PCA significantly inhibited tyrosinase activity with an IC_50_ value of 3 µM, while for MPA, a value of 30 µM was achieved. To further confirm the cosmeceutical potential of these two compounds, semisolid creams incorporating them were prepared, and their diffusion across porcine skin, used as a model for skin permeation studies and to evaluate the transdermal bioavailability, was studied. PCA was found to cross biological membranes, while MPA did not. The authors confirmed that the dissimilarity in transdermal delivery may be due to differences in the physico-chemical properties of the compounds. Unfortunately, some of these compounds displaying anti-tyrosinase activity are very sensitive to photo-oxidation and degradation, thus preventing their use in the design of cosmeceutical formulations [66].

Seo et al. [54] conducted clinical trials by exposing the forearms of 21 persons and topically applying PCA cream twice daily to evaluate the improvement in hyperpigmentation formation. The results showed that after applying the cream for seven days, there was a massive suppression of erythema formation up to 77% at the site of application, corroborating that creams with PCA can help to correct hyperpigmentation. The above findings have demonstrated that proper incorporation of these compounds in a cosmetic formulation can retain their anti-aging, anti-melanin or UV-protecting properties, encouraging further studies in the field. Caffeic acid was reported by Balupillai et al. [67] to suppress UVB-induced inflammation and photocarcinogenesis in animal models. After 10 days of continuous exposure to UV, the antioxidant levels were depleted due to the increased activity of SOD, catalase and GP, as well as the increased expression of tumor necrosis factor (TNF-α), interleukin-6 (IL-6), cyclooxygenase-2 (COX-2) and NF-κβ. The authors administered CA, both topically and intraperitoneally, to albino rats at 15 mg per mouse, which caused a remarkable decrease in the level of TNF-α, IL-6 and COX-2, while the levels of SOD, GP and catalase were restored. Furthermore, hyperplasia and other carcinogenic features caused by a constant exposure to UVB were prevented after CA administration. These comprehensive studies have demonstrated the potential of CA to be used as an anti-inflammatory, photoprotective and antiphotocarcinogenic ingredient.

In a review of Kuamar et al. [27], concerning the cosmeceutical properties of ferulic acid, it was reported that FA has the potential to absorb UV and to inhibit melanin formation through competitive inhibition of tyrosinase. These authors also highlighted the studies incorporating FA in a topical formulation containing 5% l-ascorbic acid (vitamin C) and 1% α-tocopherol (vitamin E), which showed a double photo-protection activity against the damages of long-term UV radiation. Chlorogenic acid, on the other hand, is an interesting compound with cosmeceutical significance attributed to its high ability to scavenge free radicals and to act as a UV filter [68]. These authors conducted studies to ascertain its photostability under UVA and UVB radiation. The UV protection factor was found to be nearly 92.4%, thus providing evidence for their use as UV or sunscreen filters. Very few studies have been reported concerning the anti-tyrosinase potential of chlorogenic acid [32]. The study evaluated the potential of chlorogenic acid to be used as a hyperpigmentation-correcting ingredient based on its ability to inhibit tyrosinase activity in B16 melanoma cells. The melanin content was quantified in B16 melanoma cells; after a 48-h exposure to phenolic acid at 500 μM, the melanin levels were suppressed due to a decrease in tyrosinase activity. These roles, together with other interesting biological activities reported so far, make this compound a very promising ingredient that should be fully studied and applied in cosmetic formulations.

Hyaluronic acid is an important component of skin’ extracellular matrix because it holds moisture, lubricates and also allows for collagen synthesis by upregulating transforming growth factor β (TGF-β) [5]. Inhibition of hyaluronic acid degradation tends to protect the skin and prevent aging, but very few studies have been conducted on the potential of hydroxycinnamic acids, and their derivatives, to inhibit the hyaluronidase enzyme. Murata et al. [60] isolated nine rosmarinic acid derivatives from the methanolic extract of *Meehania urticifolia* (Miq.) Makino and evaluated their hyaluronidase inhibitory activity. Rosmarinic acid had anti-hyaluronidase activity with IC_50_ of 309 µM, while its dimers, identified as rashomonic acid C, rashomonic acid D, meehanioside A, meehanioside B, meehanioside C and meehanioside D, also showed considerable anti-hyaluronidase activity with IC_50_ of 275, 183, 1049, 873, 924 and 781 µM, respectively. Aoshima et al. [44] evaluated the anti-hyaluronidase activity of caffeic acid oligomers isolated from *Clinopodium gracile*, and among the isolated compounds, clinopodic acid M revealed the strongest activity with an IC_50_ of 19 µM.

### 2.2. Anti-Inflammatory Potential

Inflammation is a complex physiological and biological response to harmful stimuli, such as pathogens, tumor cells and toxins. Inflammatory cells, such as macrophages and monocytes, usually cause increased secretion of inflammatory mediators, such as interleukins (IL 1β, IL-6, IL-8), tumor necrosis factor (TNF-α), inducible-type cyclooxygenase-2 (COX-2), prostaglandin E2 (PGE2), 5-lipoxygenase (5-LOX) and inducible nitric oxide synthase (iNOS) [69]. The expression of inflammatory mediators is usually controlled by the NF-κβ pathway, and as such, several bioactive compounds from natural sources have been reported to inhibit some steps leading to the translocation of NF-κβ to the nucleus (Table 2) [70]. Usually, exposure to UV causes the generation of ROS responsible for collagen and elastin degradation due to the increased expression of matrix metalloproteinase enzymes. This environmental stress, in combination with other factors, also causes increased expression of inflammatory mediators, such as interleukins, TNF-α, NO and COX-2, thus skin aging. Several compounds that have the ability to scavenge free radical species can be useful as antioxidants and anti-inflammatories, and the net result is a better and improved skin [71].

Atopic dermatitis (AD) is the most common skin inflammatory disorder whose physiological mechanism is not fully understood. Nevertheless, it has been associated with increased release of inflammatory mediators, such as NO, TNF-α and interleukins, as well as other irritants, causing redness, pain and edema [5]. Nonsteroidal anti-inflammatory drugs (NSAIDs) are the most common class of drugs used to suppress inflammation; they show the ability to inhibit cyclooxygenase enzyme and prevent the biosynthesis of prostaglandins, responsible for pain and inflammation, from arachidonic acid. However, long-term exposure to these drugs has been associated with various negative effects, driving the pharmaceutical industry to discover new ingredients, including natural-based ingredients, with the ability to suppress the expression of inflammatory mediators and selectively inhibit cyclooxygenase-2 enzyme. Terpenoids, polysaccharides, phenolic compounds, steroids, glycoproteins and lipid metabolites have all been reported to show prominent anti-inflammatory properties, but very few clinical studies have been carried out to identify their anti-inflammation mechanism and to determine the therapeutic doses [70]. Even though the most common compounds used to suppress atopic dermatitis are corticosteroids, moisturizers and immunosuppressive agents, some natural alternatives, such as *Spirodela polyrhiza* (L.), a pond plant [72], *Lyophyllum decastes* (Fr.) mushroom extract [73], polysaccharides (GFP) from *Grifola frondosa* (Dicks.) Gray., mushroom [74], butanol extract of *Cordyceps bassiana* (Z.Z. Li, C.R. Li, B. Huang & M.Z. Fan) [75] and ethanolic extract of *Pleurotus eryngii* (DC.) Quél. Choi et al. [76], have shown the ability to suppress the severity of the disease. Because of the broad range of health benefits ascribed to rosmarinic acid (RA), Lee et al. [77] conducted clinical studies to evaluate the potential of rosmarinic acid-based emulsions to reduce the severity of atopic dermatitis. This study was done in twenty individuals with ages comprised between five and 28 years. The RA cream was applied twice daily, for eight weeks, and the skin conditions evaluated. There was a significant improvement in the disease from erythema, dryness to other common symptoms of atopic dermatitis, thus showing the therapeutic benefits of RA as an alternative against the disease. The above findings were not able to elucidate the mechanism of anti-inflammation of RA. In another study, Jang et al. [78] induced AD-like lesions in NC/Nga mice by 0.15% 2,4-dinitrofluorobenzene (DNFB), followed by RA application (intraperitoneally at 10–50 mg/kg), which suppressed the levels of interleukin IL-4 and interferon (IFN)-γ. Recently, Tsang et al. [79] conducted in vivo studies on the potential of Pentaherbs formula (PHF), consisting of different plants, to suppress oxazolone-induced dermatitis. Chlorogenic acid was the most abundant phenolic acid, and oral ingestion of this formula reduced redness and ear swelling, while topical application had no significant effect. The level of inflammatory cytokines was suppressed, suggesting that the combined effect of the samples’ compounds, rather than individual compounds, could be responsible for the positive effect, making Pentaherbs formula an alternative for atopic dermatitis treatment.

*p*-Coumaric acid (CA), ferulic acid (FA) and their amides (diferuloylputrescine (DFP), *p*-coumaroylferuloylputrescine (CFP) and *p*-dicoumaroylputrescine (DCP)) were described by Kim, M. [80], as having interesting anti-inflammatory activity by significantly inhibiting NO production in Raw 264.7 cells. The amides were found to show better inhibition potential than the parent compounds. Many studies have reported the anti-inflammatory potential of hydroxycinnamic acids, but without identifying, or determining, the mechanism of inflammation. Bufalo et al. [81] reported some interesting results on the anti-inflammatory activity of CA. The authors confirmed that stimulation of macrophages with lipopolysaccharide, followed by exposure to CA at different concentrations, significantly suppressed nitrite production. The mechanism of action was further determined by the ability of the compound to block NF-κβ translocation to the nucleus and to prevent degradation of IκB protein, thus resulting in reduced secretion of NO. CA displays anti-inflammatory activity, but the mechanism of anti-inflammation is still not fully understood. Zhang et al. [82] induced inflammation in vivo and monitored the histological properties of the cells after exposure to CA. The authors also evaluated in vitro the amount of interleukins (IL-6 and IL-1β) and TNF-α produced or inhibited from human keratinocytes. The results showed an improvement in 12-*O*-tetradecanoylphorbol-13-acetate (TPA)-induced edema, and a remarkable decrease in IL-6, IL-1β and TNF-α levels was also observed. The authors suggested that CA might be an interesting ingredient that halts the activities of NF-κβ and finally prevents the expression of several inflammatory mediators.

Saibabu et al. [83] reviewed some of the most interesting reports on the anti-inflammatory activity of chlorogenic acid. The authors stated that this compound displays anti-inflammatory potential mainly by suppressing the expression of inflammatory mediators, downregulation of the NF-κB pathway and downregulation of iNOS, COX-2 and PGE2.

Recently, Alam et al. [84] reviewed some of the most interesting anti-inflammatory studies, both in vivo and in vitro, reported on hydroxycinnamic acids and derivatives, such as ferulic acid and its salt, *p*-coumaric acid, caffeic acid and its phenethyl, butyl and octyl esters, as well as chlorogenic acid. The mechanism of anti-inflammation was attributed to the ability of these compounds to: (1) inhibit the expression of TNF-α; (2) inhibit the NF-κβ pathway; (3) suppress the expression of COX-2, iNOS, VCAM-1 and ICAM-1; (4) prevent the phosphorylation and activation of NF-κβ-inducing kinase/Iκβ kinase (NIK/IKK) and mitogen-activated protein kinase (MAPKs); (5) inhibit the COX-1 and COX-2 enzymes; (6) block the activation of the AP-1 transcription factor; and (7) suppress the secretion of PGE2.

### 2.3. Antimicrobial Activity

Parabens are synthetic alkyl esters of *p*-hydroxybenzoic acid that are common preservatives in personal care products and pharmaceuticals, preventing the growth of bacteria thanks to their broad spectrum of antimicrobial activity. However, some of their butyl and propyl esters have been discovered to cause reproductive defects [106]. Their use is now facing some constraints, with the cosmetic industry starting to exclude this ingredient from their products [107]. In this context, bioactive compounds from natural sources can become viable alternatives based on their antimicrobial activity. Phenolic acids, such as ferulic and caffeic acids and their derivatives, are known to cause disruption of bacterial and fungal cell membrane, allowing the leakage of the cytoplasmic membrane and, finally, cell death [108]. Martins et al. [109] reviewed some of the most interesting results produced so far on the anti-*Candida* potential of hydroxycinnamic acids and derivatives, such as caffeic acid, chlorogenic acid, *m*-coumaric acid (2-hydroxycinnamic acid), *o*-coumaric acid (3-hydroxycinnamic acid), *p*-coumaric acid (4-hydroxycinnamic acid) and ferulic acid. The authors concluded that, among the large classes of phenolic compounds, phenolic acids and their derivatives showed very interesting results and that the mechanism of action is related to their ability to disrupt the fungal cell membrane.

Khatkar et al. [110] evaluated the inhibitory power of amide, anilide and ester derivatives of *p*-coumaric acid against *Staphylococcus aureus*, *Bacillus subtilis*, *Escherichia coli*, *Aspergillus niger* and *Candida albicans*, to confirm the viability of their use as preservatives in cosmeceutical formulations. The results showed that all derivatives of *p*-coumaric acid were very effective and displayed even better activity than the control, being interesting for the replacement of some of the currently-used cosmetic preservatives. Karunanidhi et al. [111] investigated the antibacterial activity of chlorogenic acid against nine clinical isolates of *Stenotrophomonas maltophilia* by evaluating their minimum inhibitory concentration (MIC) and minimum bactericidal concentration (MBC) through biofilm and disk diffusion assays. The compound was found to have a MIC and MBC of 8–16 μg/mL and 16–32 μg/mL, respectively; while the bacterial inhibition zones were between 17 and 29 mm. These studies confirm chlorogenic acid as an antibacterial ingredient, even though the mechanism of action has not been elucidated. The antibacterial activities of caffeic acid and chlorogenic acid were evaluated by Jeong et al. [112], by using the disc diffusion method against *Staphylococcus aureus*, *Staphylococcus epidermidis*, *Bacillus cereus* and *Pseudomonas aeruginosa*. Both compounds displayed interesting antibacterial activity. A survey of studies conducted on the potential of hydroxycinnamic acids and their derivatives to inhibit the growth of microbial pathogens is shown in Table 3.

The mechanism of antimicrobial activity displayed by these compounds has been largely attributed to their ability to disrupt cell membranes and cause cytoplasmic leakage, both in Gram-negative and Gram-positive bacteria. These compounds also inhibit enzymes involved in the pathway leading to ergosterol synthesis in *Candida* species, induce apoptosis, disrupt the membrane integrity and membrane potential, causing the blockage of nutrient flow, and allow the leakage of nucleotides [108,113].

## 3. Challenges in the Use of Hydroxycinnamic Acids in Topical Formulations

### 3.1. Microencapsulation

Compounds from natural sources have medicinal properties that can support the design of innovative cosmeceutical formulations. Nevertheless, most of these compounds face limitations, such as poor stability, increased oxidation, degradation and poor permeability across biological membranes [29]. To overcome these constraints, bioactive compounds have been protected by several polymeric materials, through microencapsulation techniques, to avoid degradation and allow a sustained and gradual release at target sites [3]. The pharmacological and cosmeceutical potential displayed by hydroxycinnamic acids also needs to surpass these challenges to be fully exploited in cosmetic formulations. Attention is now focused on finding effective microencapsulation methods that will optimally encapsulate the bioactive materials ensuring the crossing of biological membranes.

Casanova et al. [29] prepared microparticles containing rosmarinic acid using chitosan and modified chitosan as the encapsulating materials. Rosmarinic acid has vital biological properties, being an interesting ingredient for cosmetic formulations, but its use is limited due its poor stability, degradation and slow transport across biological membranes. The authors employed a spray drying technique to produce the microcapsules, and their release profile was analyzed in aqueous and coconut oil media. Encapsulation yields and particle size were 42.6% and 39.8% and 4.2 μm and 7.7 μm for chitosan and modified chitosan particles, respectively, and the results showed that the chitosan particles displayed a slower release in water, while those in oil were more sustained. Furthermore, Biswick et al. [135] explained that due the poor stability of caffeic acid towards solar radiation and oxidation, microcapsules were prepared using zinc hydroxide nitrate as a matrix to prevent degradation and ensure a sustainable release compatible with cosmeceutical applications. The prepared microparticles were able to withstand both UVA and UVB radiation, indicating that this technology can be applied in the design of sunscreen cosmetic formulations.

Due to the unique properties exhibited by chitosan, it was selected by Lee et al. [136] for encapsulating hydroxycinnamic acids, such as caffeic acid, ferulic acid and sinapic acid. The idea was to develop a conjugate made up of chitosan and the phenolic acids to evaluate and compare their bioactive properties. The antioxidant activity was screened by DPPH and 2,2′-azino-bis (3-ethylbenzothiazoline-6-sulphonic acid (ABTS) assays, and the results indicated that chitosan conjugated with hydroxycinnamic acids displayed higher radical-scavenging activity than the unmodified form. The results also showed that the conjugated chitosan inhibited bacterial growth, suggesting that this application can be useful in formulations preventing bacterial growth, thus, increasing shelf life. *Candida*, a common skin microflora, was studied by Panwar et al. [137], which reported the potential of encapsulated ferulic acid against *C. albicans*. Ferulic acid was encapsulated into chitosan with an encapsulation efficiency and particle size diameter of 56.45% ± 2.47% and 52.54 ± 0.22 nm, respectively. The stability and integrity of the phenolic acid after encapsulation were also studied; the nanoparticles showed activity against *C. albicans* biofilm formation, confirming the integrity of ferulic acid in the microencapsulated form. The results showed that the microencapsulation procedures can provide efficient tools to support the development of cosmetic formulations against fungal infection.

Several technologies can be used for the encapsulation of bioactive compounds [138]. The choice of the encapsulating material depends on several factors, including the properties of the target bioactive compound, the application to be developed and the intended release profile. Recently, Ignatova et al. [139] encapsulated caffeic acid using poly (3-hydroxybutyrate) (PHB) and poly ethylene glycol (PEG). The nanoparticles prepared by electrospinning were evaluated for antibacterial activity against *S. aureus* and *E. coli*, as well as for their release profile. The results showed that 95.4% and 97.0% of caffeic acid were released after 24 h from PHB and PEG, respectively. Free and nanoencapsulated CA displayed antibactericidal activity against *S. aureus* after 24 h exposure at 1800 μg/mL. Ouimet et al. [28] showed that the encapsulated ferulic acid presented a slow release over time, it being necessary to work on alternative methods to ensure a quicker release adapted to topical delivery. These authors reported that the incorporation of ethylene glycol with the encapsulating material could allow a faster degradation of the polymeric matrix and, therefore, a faster release. Complete release of ferulic acid was achieved after a few days, and the antioxidant activity was also found to be higher. Furthermore, Vashisth et al. [140] isolated and characterized ferulic acid from the *Parthenium hysterophorus* L. plant, further nanoencapsulated by electrospinning. Poly lactic-co-glycolic acid (PLGA) and polyethylene oxide (PEO) were employed as encapsulating materials at a PLGA:PEO ratio of 1:1 using dichloromethane/dimethylformamide (4:1, *v*/*v*) as the solubilizing medium. The free and encapsulated ferulic acid were evaluated for DPPH radical scavenging activity. The antioxidant activity of the encapsulated ferulic acid was found to be 59%, while the isolated compound gave 41%, thus suggesting that encapsulated ferulic acid preserved the antioxidant activity.

Most of the systems used to microencapsulate bioactive compounds for topical application face limitations, and the choice of the encapsulating system often depends on the particle size of choice, the physical and chemical properties of the encapsulating material, the intended release profile and the cost of production. Even though the active ingredient can be efficiently encapsulated, there is still a continuous search for new methods and the development of a hybrid system to overcome some of the limitations associated with the process of microencapsulation [141]. Another major drawback in the use of the microencapsulation system in personal care products involves properly evaluating the release profile of the active ingredient from the encapsulating material, as well as skin permeation studies. Furthermore, the behavior of the microparticles produced needs to be further studied to be able to understand the mechanism of diffusion into the skin and how the microparticles interact with the lipid component of the stratum corneum. Hence, conducting more studies will further allow for a better understanding of the choice of encapsulating system and the polymeric material.

### 3.2. Skin Permeation Studies

The barrier properties of the stratum corneum of the skin have made it difficult for transdermal penetration of some ingredient in cosmetic formulation. The stratum corneum offers a barrier against the entry of foreign materials, pathogens and loss of moisture [142]. Physicochemical parameters, such as the molecular weight and lipophilic/hydrophilic balance, have been reported to affect the permeability of compounds in cosmetic formulation through skin [143]. Conducting in vitro permeation studies using humans and animals is important to fully understand the permeation profile of these topically-applied chemicals. However, using human samples is very expensive and is subject to ethical evaluation, and that has led to the increased search for an alternative artificial skin diffusion apparatus [143,144]. For an in vitro skin apparatus like the Strat-M™ (Merck Millipore, Billerica, MA, USA), PermeGear inline diffusion cells (PermeGear Inc., Bethlehem, PA, USA) have been developed to mimic the skin in a Franz-type diffusion cell, while epidermal full thickness (EFT) human skin cultures (MatTek, Ashland, MA, USA) can also be used to validate the penetration through skin for various hydroxycinnamic acids and its derivatives.

The skin permeation studies of hydroxycinnamic acids have not been fully studied, but among the ones available in the literature, only ferulic acid seems to be the most widely reported. In vitro experiments were conducted by Thitilertdecha et al. [145] using a pig skin model with cosmetic formulation containing phenolic compounds, but none of the hydroxycinnamic acids were able to penetrate the skin in an amount that could be quantified after 3 h of exposure. The authors suggested that the use of a potential skin penetration enhancer, like propylene glycol, can increase skin penetration of these compounds, but the use of penetration enhancers is subject to controversies and still lacks regulatory approval [146].

In vitro skin permeation studies of a gel containing coumaric, caffeic and ferulic acids were conducted by Zilius et al. [147] using Bronaugh-type diffusion cells. The results showed that after a 24-h exposure of the gel to the skin cells, the epidermis was found to contain 5% coumaric and ferulic acids and 4% coumaric and 6% ferulic acid in the dermis, while no traces of caffeic acid were found. The authors report that the differences in penetration might be associated with the variation in the lipophilicity of the tested compounds, as well as the composition of the cosmetic vehicle used.

Other authors prepared an aqueous vehicles with ferulic acid (FA) and its skin permeation potential evaluated using porcine skin because of its physiological similarity to human skin [148]. Ferulic acid was found to permeate the skin with the flux; a term used to denote the amount of compound that traverses across a unit area of the skin per unit time reported to be between 0.48 ± 0.10 and 1.16 ± 0.30 nmol/cm^2^/h at pH 6 and pH 9, respectively. The result suggests that this compound exhibits penetration ability across the skin, and the pH of the vehicle does not significantly affect the penetration profile of this compound. Overall, ferulic acid seem to be one of the hydroxycinnamic acids with the most in vitro permeation studies reported [149,150,151,152]. Hence, more studies need to be conducted on other hydroxycinnamic acids.

## 4. Concluding Remarks

Hydroxycinnamic acids have proven to be promising ingredients for cosmeceutical purposes due to their interaction with several biochemical processes in the skin, namely antioxidant augmentation, UV filtering, suppression of cytokine levels, melanin inhibition, promotion of collagen synthesis, suppression of collagenase, photoprotection and anti-elastase activity. Despite the interesting results, herein surveyed for hydroxycinnamic acids and their derivatives, both in vitro and in vivo, their cosmeceutical potential is still not well established due to several limitations. In this context, microencapsulation techniques emerged as very promising techniques to protect these bioactives from oxidation and degradation, allowing also for their gradual release at target sites.

However, very few studies report clinical trials, kinetics studies and the ability of the nanoparticles to cross biological membranes in order to fully confirm the cosmeceutical benefits. Among the hydroxycinnamic acids, *p*-coumaric acid and ferulic acid seem to be the most widely-studied forms taking into consideration in vitro, in vivo and skin permeation studies to predict their transdermal bioavailability, as well as clinical trials in humans. Most studies dealing with the cosmeceutical potential of hydroxycinnamic acids revealed contrasting results. This was associated with the fact that the concentration at which a compound display activity might vary according to the used bioassay, making it difficult to come to an effective resolution congregating all of the reported bioactivities.

From the bioactive properties reported for hydroxycinnamic acids and their derivatives presented in Table 1, Table 2 and Table 3, most of the therapeutic concentrations present high values. When these compounds eventually become incorporated in a base cosmetic cream (1%–5%), clinical studies, both in vivo and in vitro, need to be conducted in order to establish the cosmeceutical potential of these compounds. The mechanism of action of the ascribed bioactive properties is mostly known, but more skin permeation tests, namely the ones using mammalian dermal cells, should be carried out to demonstrate the formulation’ ability to cross the membrane and be absorbed, while keeping membrane integrity. All of these mentioned limitations, when properly addressed, will evidence the cosmeceutical significance of hydroxycinnamic acids and their derivatives.

## 5. Future Perspectives

The cosmetic industry is always in search of new and improved multifunctional ingredients from natural sources. Recently, several benefits have been addressed to hydroxycinnamic acids, namely anti-wrinkling, photoprotective, anti-inflammatory and antimicrobial properties and depigmenting potential. To be able to fully exploit these compounds in cosmeceutical formulations, some measures should be taken: (i) optimize extraction techniques to maximize the recovery of hydroxycinnamic acids from natural sources; (ii) perform tests directed to the final application to validate their effectiveness in cosmetic matrices; (iii) explore microencapsulation techniques with different polymeric materials to modulate the release and provide protection against environmental factors; (iv) conduct skin permeation studies to evaluate the topical availability of the bioactive compounds incorporated in the formulations, as well as clinical studies fulfilling ethical principles for studies involving human subjects, to confirm the cosmeceutical potential of these compounds.

## Figures and Tables

**Table 1:** Anti-aging and anti-tyrosinase activities reported for hydroxycinnamic acids and their derivatives.

Compound	Source	Bioactivity	Effects	Reference
Ascorbyl-3-*p*-coumarate; Ascorbyl-2-*p*-coumarate	Commercial	Antityrosinase	At 100 µM, decreased melanin content by 65% and 59%, respectively.	[25]
Ascorbyl-3-*p*-coumarate; Ascorbyl-2-*p*-coumarate	Commercial	Anti-collagenase	At 100–300 µM, they promoted collagen release by 120%–144% and 125%–191%, respectively.	[25]
Caffeoyl-amino acidyl-hydroxamic acid	Synthesized	Antityrosinase	At 100 μM, displayed anti-tyrosinase activity.	[43]
Caffeic acid oligomers	*Clinopodium gracile* (Benth.) Kuntze	Anti-hyaluronidase	From 19–1000 μM, compounds showed up to 50% anti-hyaluronidase activity.	[44]
Chlorogenic acid	Commercial	Antityrosinase	At 500 μM after 48-h exposure to B16 melanoma cells, melanin levels were suppressed.	[32]
Dietary phenolic acids	Commercial	Antityrosinase	*p*-Coumaric acid 22.86 ± 2.1, caffeic acid 43.09 ± 2.3 and ferulic acid 51.85 ± 1.7 μM were responsible for 30% inhibition of tyrosinase activity.	[45]
Dietary phenolic acids	Commercial	Antityrosinase	Caffeic acid 24.1 + 6.2 and ferulic acid >30 μM caused 30% inhibition f tyrosinase.	[46]
Ferulic and caffeic acids	Commercial	Anti-collagenase	Ferulic (15–30 µM) and caffeic (3.75–30 µM) suppressed UVA-induced MMP-1 activity.	[47]
Ferulic and caffeic acids	Commercial	Photoprotection	Both compounds at 200 µL offered protective activity to UVB-induced skin erythema.	[48]
Ferulic acid	Commercial	Anti-collagenase	FA applied topically at 0.01, 0.05–1 mg/site/mouse, significantly suppressed the expression of MMP-2 and MMP-9.	[49]
Hydroxycinnamic amides	Synthesized	Antityrosinase	At 0.185–475 µM, all nine derivatives significantly inhibited tyrosinase activity up to 50%.	[50]
*N*-Hydroxycinnamoyl amides	Synthesized	Antityrosinase	All investigated amides significantly inhibited tyrosinase activity.	[51]
*p*-Coumaric acid	Commercial	Antityrosinase	At 10 µg/mL, showed a higher tyrosinase activity inhibition than arbutin, but comparable to kojic acid.	[52]
	*Oryza sativa* L.	Antityrosinase	Reduced MITF and tyrosinase mRNA expression by 73% and 82%, respectively.	[53]
	Commercial	Antityrosinase	Inhibited hyperpigmentation up to 77% in human skin.	[54]
	Commercial	Anti-collagenase	At 30 µg/mL, inhibited MMP-1 expression from dermal fibroblasts.	[55]
*p*-Coumaric acid, methyl *p*-coumarate	Commercial	Antityrosinase	*p*-Coumaric acid, methyl *p*-coumarate at 3 µM and 30 µM caused 50% tyrosinase inhibition.	[56]
Rosmarinic acid	*Salvia officinalis* L.	Antityrosinase	At 10 μM, tyrosinase activity was inhibited by 20%.	[57]
Rosmarinic acid	*Prunella vulgaris* L.	Photoprotection	At 0.9–18 mg/L, UVA-induced changes in human keratinocytes cells were suppressed.	[58]
	*Rosmarinus officinalis* L.	Photoprotection	Oral administration of rosmarinic acid suppressed cutaneous alterations in vivo due to UVA exposure.	[59]
Rosmarinic acid derivatives	*Meehania urticifolia* (Miq.) Makino	Anti-hyaluronidase	Between 183 and 1049 μM, compounds showed up to 50% anti-hyaluronidase activity.	[60]
Rosmarinic acid, methyl rosmarinate	*Rabdosia serra* (Maxim.) Y.N. Lee	Antityrosinase	At 0.4 mM, rosmarinic acid and methyl rosmarinate inhibited tyrosinase activity by 19.80% and 37.10% respectively.	[61]
Rosmarinic acid methyl ester	*Origanum vulgare* L.	Antityrosinase	At 20 μg/mL, the expression of MITF, tyrosinase, TRP-2 and TRP-1 was downregulated.	[62]

MMP-1: matrix metalloproteinase-1, MMP-2: matrix metalloproteinase-2, MMP-9: matrix metalloproteinase-9, MITF: microphthalmia-associated transcription factor, TRP-1: tyrosinase-related protein-1, TRP-2: tyrosinase-related protein-2.

**Table 2:** Anti-inflammatory activity reported for hydroxycinnamic acids and their derivatives.

Compound	Source	Effect	Reference
1-*p*-Coumaroyl β-d-glucoside	*Salix hulteni* L.	Up to 400 µM suppressed TNF-α and IL-1β levels, reduced iNOS and COX-2 expression and inhibited Iκβ degradation.	[85]
3,4,5-Trihydroxycinnamic acid	Commercial	At 100 µM, it suppressed NO production up to 70% and reduced Iκβ degradation.	[86]
Acetyl-caffeic acid–1-piperonylpiperazine	Synthesized	At 20 μM, up to 60%–70% of NO was suppressed and NF-κβ activation inhibited.	[87]
Caffeic acid	Commercial	At 10–200 μg/mL, IL-8, IL-1β, IL-6 and TNF-α levels were suppressed, IκBα degradation and p65 phosphorylation inhibited.	[88]
	Commercial	At 10 μg/mL, it suppressed NO levels, blocked NF-κβ translocation and prevented IκB-α degradation.	[81]
Caffeic acid phenethyl ester (CAPE)	Commercial	At 1 µM, COX-1 and IL-1β expression was suppressed.	[89]
Caffeic acid derivatives (methyl, ethyl, butyl)	Commercial	At 21.4, 11.9 and 8.4 µM, the derivatives inhibited NO levels up to 50%.	[90]
Caffeic acid methyl vanillate ester	Synthesized	At 15 μM, it suppressed NO levels and inhibited TNF-α, COX-2 and ICAM-1 expression.	[91]
Chlorogenic acid	Commercial	0.5–100 μmol/L of CGA suppressed the expression of NF‑κB, p50 and IKKα/β.	[92]
	Commercial	Intraperitoneally at 2.5–50 mg/kg, it suppressed TNF-α, IL-1β and IL-6 release by inhibiting the TLR4-mediated NF-κβ signaling pathway.	[33]
	Commercial	At 20 μM, levels NO, IL-1β, TNF-α and IL-6 were suppressed and the expression of COX-2 and iNOS reduced.	[93]
	*Cymbopogon citratus* (DC.) Stapf	At 140 μg/mL, the level of NO was significantly suppressed.	[94]
	Commercial	Up to 20 μM of CGA reduced the expression of IL-1β and COX-2.	[95]
Cinnamic acid, glucuronated and methylated derivatives	Synthesized	NO levels were suppressed significantly at 224 ± 16 μM.	[69]
Ferulic	Commercial	FA topically and intraperitoneally inhibited the expression of TNF-α and IL-6.	[96]
Hydroxycinnamic amides	Corn bran	All four amides evaluated inhibited NO level and dose-dependently suppressed iNOS expression.	[97]
*p*-Coumaric acid, glucuronated and methylated derivative	Synthesized	NO levels were suppressed significantly at 442 ± 33 μM.	[69]
*N*-(*p*-Coumaroyl) tryptamine	*Zea mays* L.	Up to 40 µM suppressed TNF-α, NO, PGE2, IL-1β, iNOS and COX-2 expression and prevented JNK/c-Jun and Akt phosphorylation.	[98]
*p*-Coumaric	Commercial	Suppressed TNF-α levels in vivo at 100 mg/kg body weight in arthritis-induced rats.	[99]
Rosmarinic acid	*Cordia Americana* (L.) Gottschling & J.S.Mill.	At 36.03 µg/mL, TNF-α levels were inhibited up to 36.75% ± 1.55%, and MAPK was inhibited up to 50% at 1.16 ± 0.13 µg/mL.	[100]
	Commercial	At 2.75 μM expression of IL-6 and IL-8 was suppressed.	[101]
	Commercial	At 1 µg/mL, TNF-α levels were reduced and iNOS expression suppressed.	[102]
	*Prunella vulgaris* L.	At 2.67 μM, PGE2 and NO production was inhibited by 15% and 17%, respectively.	[103]
	Commercial	TNF- α, IL-6 and IL-1β levels were suppressed after administration of 5, 10 and 20 mg/kg of rosmarinic acid/mice weight.	[104]
	*Prunella vulgaris* L.	At 66 μg/mL, PGE2 production was suppressed by 72%	[80]
Trans-caffeic acid	*Cordia sinensis* Lam.	At 100 mg/kg, it suppressed carrageen-induced paw edema in rat by 50%	[105]

COX-2: cyclooxygenase-2, ICAM-1: intercellular adhesion molecule-1, IKK: Iκβ kinase, IL-1β: interleukin 1β, IL-6: interleukin 6, IL-8: interleukin 8, iNOS: inducible nitric oxide synthase, MAPK: mitogen-activated protein kinase, NF-κβ: nuclear factor-κB, NO: nitric oxide, PGE2: prostaglandin E2, TLR4: Toll-like receptor 4, TNF-α: tumor necrosis factor α.

**Table 3:** Antimicrobial activity reported for hydroxycinnamic acids and their derivatives.

Compound	Source	Microorganism	Effect	Reference
2-Coumaric acid	Synthesized	*Mycobacterium tuberculosis*.	MIC value of 122 µM	[114]
3,4-Dialkoxy caffeic acids	Synthesized	*Staphylococcus aureus*, *Corynebacterium diphtheria*, *Escherichia coli*, *Klebsiella pneumonia*, *Salmonella typhi*.	GI 100 μg/mL	[115]
5-*O*-caffeoylquinic acid	*Coffea robusta* L.Linden	*S. aureus*, *Streptococcus mutans*.	2.7–6.3 mg/mL	[116]
	Commercial	*Escherichia coli*, *Staphylococcus aureus*, *Enterococcus faecium*, *Proteus vulgaris*, *Pseudomonas aeruginosa*, *Klebsiella pneumoniae* and *Candida albicans*.	MIC 5–10 mg/mL	[117]
Caffeic and cinnamic acid ester	Synthesized	*Candida albicans* biofilm.	MIC 32 µg/mL	[118]
Caffeic, chlorogenic, *o*-coumaric, *p*-coumaric acid	Commercial	*E. coli*, *S. aureus*, *Salmonella typhimurium*, *Lactobacillus rhamnosus*.	MIC 125–1000 μg/mL	[119]
Caffeoylquinic acids	*Artemisia absinthium* L.	*S. aureus*, *E. faecalis*, *E. coli*, *C. albicans*, Methicillin-resistant *S. aureus*, *Bacillus cereus*.	MIC 32–256 μg/mL	[120]
Caffeoylquinic acids	Prunus mume seeds	*S. aureus*, *E. coli*, *Salmonella enterica*, *Vibrio parahaemolyticus*, *C. albicans*, *Saccharomyces cerevisiae*, *Aspergillus niger*.	MIC 10–250 μg/mL	[121]
Chlorogenic acid	Synthesized	*S. aureus*, *Streptococcus pneumoniae*, *Bacillus subtilis*, *E. coli*, *Shigella dysenteriae Salmonella Typhimurium*.	MIC 20–80 μg/mL	[113]
Chlorogenic, rosmarinic, sinapic and ferulic acid	Commercial	*Campylobacter jejuni*, *Campylobacter coli*.	MIC 4.9–313 μg/mL	[122]
Ferulic acid	*Halimodendron halodendron* (Pall.)	*Agrobacterium tumefaciens*, *E. coli*, *Pseudomonas lachrymans*, *Xanthomonas vesicatoria*, *B. subtilis*, *S. aureus*, *Staphylococcus haemolyticus*. *C. albicans* and *Magnaporthe oryzae*.	MIC 28.1–149.7 μg/mL	[123]
	Commercial	*P. aeruginosa*, *E. coli*, *L. monocytogenes*, *S. aureus* biofilm formation.	MBC 500–5000 μg/mL	[124]
	Commercial	*Bacillus cereus* and *Pseudomonas fluorescens* single- and dual-species biofilms.	MIC 500 μg/mL	[125]
Ferulic acid esters	Synthesized	*Escherichia coli*, *Klebsiella pneumoniae*, *Staphylococcus aureus*, *Enterococcus faecalis*, *Candida albicans*, *Candida krusei*, *Candida parapsilosis*.	MIC 8–1024 μg/mL	[126]
Ferulic acid, *p*- coumaric acid	Commercial	*Bacillus cereus*, *Micrococcus flavus*, *S. aureus*, *Listeria monocytogenes*, *E. coli*, *Enterobacter cloacae*, *P. aeruginosa*, *S. typhimurium*, *C. albicans*.	MIC 0.01–0.04 mg/mL	[127]
*o*-Coumaric, *m*-coumaric, *p*-coumaric acid	Commercial	*C. albicans*, *Candida parapsilosis*, *Candida glabrata*, *Candida tropicalis*, *Candida krusei*, *Candida lusitaniae*, *Cryptococcus neoformans*.	GI 5.9%–99.9%	[128]
*p*-Coumaric acid	Commercial	*S. aureus*, *Streptococcus pneumoniae*, *B. subtilis*, *E. coli*, *Shigella dysenteriae*, *S. typhimurium*.	MIC 10–80 μg/mL	[129]
*p*-Coumaric acid derivatives	Synthesized	*S. aureus*, *B. subtilis*, *E. coli*, *C. albicans*, *Aspergillus niger*.	MIC 0.68–1.93 μM/mL	[110]
Rosmarinic acid	*Zostera marina* L.	*Pantoea agglomerans*, *Stenotrophomonas maltophilia*, *Klebsiella* sp., *Streptomyces* sp.	MIC 1 mg/mL	[130]
	*Rosmarinus officinalis* L.	*S. aureus*.	MIC 5 µg/mL	[131]
Rosmarinic acid, methyl rosmarinate	*Hyptis atrorubens* Poit.	*Staphylococcus epidermidis*, *Stenotrophomonas maltophilia*, *Enterococcus faecalis*, *Staphylococcus lugdunensis*, *P. aeruginosa*, *Corynebacterium*, *Mycobacterium smegmatis*, *Staphylococcus warneri*.	MIC 0.3–2.5 mg/mL	[132]
Sinapic acid	*Brassica juncea* L.	*B. subtilis*, *E. coli*, *Listeria innocua*, *Listeria monocytogenes*, *Pseudomonas fluorescens*, *S. aureus*, *Lactobacillus plantarum*.	MIC 0.2–0.7 g/L	[133]
Trans-cinnamaldehyde, *p*-coumaric, ferulic acid	Commercial	*E. coli* biofilm.	0.25%–0.5% concentration	[134]

GI: growth inhibitory activity, MBC: minimal bactericidal concentrations, MIC: minimum inhibitory concentration.
